# Supplementary material for: An evaluation of fusion partner proteins for paratransgenesis in Asaia bogorensis
Source: PLoS One. 2022 Sep 1;17(9):e0273568. doi: 10.1371/journal.pone.0273568 (PMC9436115; doi:10.1371/journal.pone.0273568)
Supplement: S1 Table — Restriction sites are underlined. (PDF) [file pone.0273568.s001.pdf]

S1 Table: Oligonucleotides and synthetic dsDNA fragments used in this study. Restriction sites are underlined.

**Primers Used**	**Nucleotide Sequence 5’ to 3’**	**Purpose**
Secreted Scorpine Vector F	AACCGCGCCCGGCAGT	Directional cloning of Hyp4s plasmid backbone for Myc affinity tag and restriction sites insertion into Hyp4s to replace the ‘*phoA* gene using Gibson Assembly.
Secreted Scorpine Vector R	CTGCAGGGCGAACCGCC	Directional cloning of Hyp4s plasmid backbone for Myc affinity tag and restriction sites insertion into Hyp4s to replace the ‘*phoA* gene using Gibson Assembly.
Prot fusion seq F	GCGGTTCGCCCTGCA	PCR and sequence verification of fusion partner constructs
Prot fusion seq R	ACAGCAAAAAAACCACCCGG	PCR and sequence verification of fusion partner constructs
TrxA F	GATCTGAACCCTAGGATGAGCGATAAAATTATTCACCT	Directional cloning of thioredoxin protein into pHyp4s.Myc vector using Gibson Assembly
TrxA R	GCGCGGTTAGGCGCGCCTTAGGCCAGGTTAGCGTC	Directional cloning of thioredoxin protein into pHyp4s.Myc vector using Gibson Assembly
GST F	GATCTGAACCCTAGGATGTCCCCTATACTAGGTT	Directional cloning of glutathione S-transferase protein into pHyp4s.Myc vector using Gibson Assembly
GST R	GCGCGGTTAGGCGCGCCTTATTTTGGAGGATGGTCG	Directional cloning of glutathione S-transferase protein into pHyp4s.Myc vector using Gibson Assembly
MBP F	GATCTGAACCCTAGGATGAAAATCGAAGAAGGTAAACTGG	Directional cloning of maltose binding protein into pHyp4s.Myc vector using Gibson Assembly
MBP R	GCGCGGTTAGGCGCGCCTTAAGTCTGCGCGTCTT	Directional cloning of maltose binding protein into pHyp4s.Myc vector using Gibson Assembly
6xHis top strand	CTAGGATGCACCATCATCATCACCACTAAGG	Combined with 6xHis bottom strand for directional cloning of 6xHis affinity tag into pHyp4s.Myc vector

6xHis bottom strand	CGCGCCTTAGTGGTGA TGATGATGGTGCATC	Combined with 6xHis top strand for directional cloning of 6xHis affinity tag into pHyp4s.Myc vector
**gBlocks™ Gene Fragments Used**	**Nucleotide Sequence 5’ to 3’**	**Purpose**
Myc tag fusion construct	AGCGGCGGTGGCGGTT CGCCCTGCAGGGAACA GAAACTGATCTCGGAG GAAGATCTGAACCCTA GGTTCTTATGGCGCGCC TAACCGCGCCCGGCAG TGAATTTTCGCTGCCGG GTGGTTTTTTTG	Directional cloning of Myc affinity tag and restriction sites into Hyp4s to replace the *‘phoA* gene using Gibson Assembly.
