# Supplementary material for: An evaluation of fusion partner proteins for paratransgenesis in Asaia bogorensis
Source: PLoS One. 2022 Sep 1;17(9):e0273568. doi: 10.1371/journal.pone.0273568 (PMC9436115; doi:10.1371/journal.pone.0273568)
Supplement: S1 Raw image — The membrane was visualized on an Odyssey FC dual mode imaging system (LI-COR) using the 800 nm infrared fluorescent detection channel for 2 min. Image Studio Software 5.0 (LI-COR) was used for blot visualization. SF2.1, wild-type Asaia; +, pIT2-scFv Myc positive control; MBP*, MBP diluted to a final OD600 of 10. (PDF) [file pone.0273568.s002.pdf]

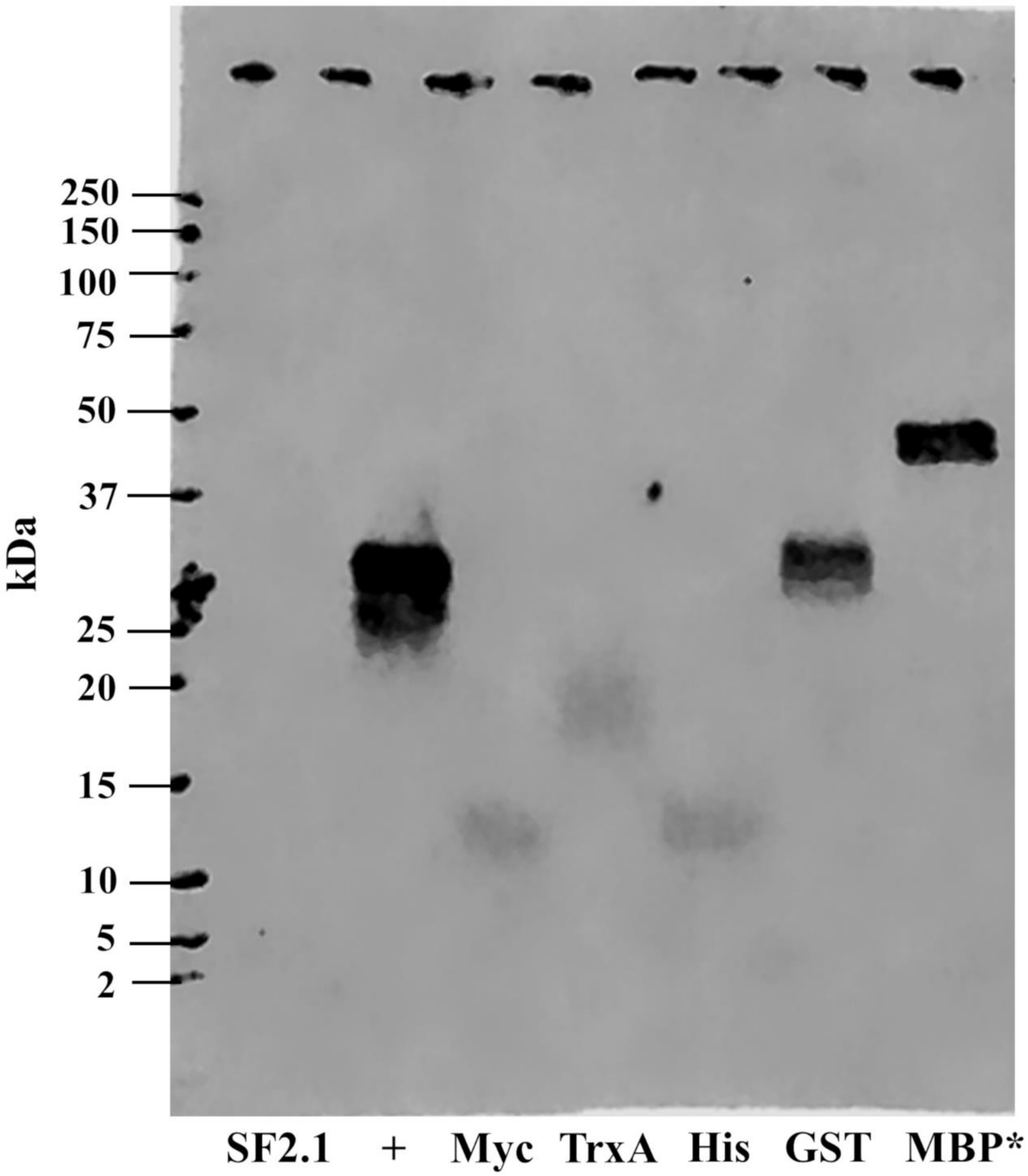

S1 Raw Image. Original uncropped western blot corresponding to Fig 2C in the manuscript, marked in the same manner. The membrane was visualized on an Odyssey FC dual mode imaging system (LI-COR) using the 800 nm infrared fluorescent detection channel for 2 min. Image Studio Software 5.0 (LI-COR) was used for blot visualization. SF2.1, wild-type *Asaia*; +, pIT2-scFv Myc positive control; MBP*, MBP diluted to a final OD_{600} of 10.
